# Comparative genomics of *Acinetobacter baumannii* from Egyptian healthcare settings reveals high-risk clones and resistance gene mobilization

**DOI:** 10.1186/s12879-025-11185-x

**Published:** 2025-06-11

**Authors:** Salma Salem, Dina Osama, Nehal Adel Abdelsalam, Ahmed H. Shata, Shaimaa F. Mouftah, Mohamed Elhadidy

**Affiliations:** 1 Center for Genomics, Helmy Institute for Medical Sciences, Zewail City of Science and Technology, Giza, Egypt; 2 Biomedical Sciences Program, University of Science and Technology, Zewail City of Science and Technology, Giza, Egypt; 3 Department of Microbiology and Immunology, Faculty of Pharmacy, Heliopolis University, Cairo, Egypt; 4 Department of Bacteriology, Mycology and Immunology, Faculty of Veterinary Medicine, Mansoura University, Mansoura, Egypt

**Keywords:** *Acinetobacter baumannii*, Antimicrobial resistance, Mobile genetic elements, Egypt, International clones

## Abstract

**Background:**

*Acinetobacter baumannii* (*A. baumannii*) has emerged as a major public health threat in low- and middle-income countries (LMICs), particularly in Egypt, due to its remarkable ability to acquire and transfer resistance genes, as highlighted in the WHO bacterial Priority Pathogens List 2024 classification. This pilot study aimed to characterize 18 *A. baumannii* isolates from Egyptian healthcare settings, focusing on clonal lineages, antibiotic resistance determinants, horizontal gene transfer potential, and the presence of virulence factors and chromosomal mutations.

**Methods:**

Antimicrobial susceptibility testing was performed to determine resistance profiles using minimum inhibitory concentrations. Whole-genome sequencing was used to identify *β*-lactamase, carbapenemase, and other antibiotic resistance genes (ARGs), as well as mobile genetic elements (MGEs). Clonal relationships among isolates were assessed via core genome multilocus sequence typing (cgMLST).

**Results:**

Phenotypic analysis revealed that 72% of the isolates were extensively drug-resistant (XDR), exhibiting resistance to all tested antibiotics except colistin. Clonal diversity analysis identified 11 Oxford sequence types (STs), including two novel STs (ST3309^OXF^ and ST3321^OXF^), and six international clonal (IC) groups, with IC2 being the most prevalent. Additionally, eight Pasteur STs were detected, with ST570^PAS^ being the most frequent. The cgMLST analysis showed that two Egyptian ST570^PAS^ isolates clustered with a strain from Saudi Arabia, suggesting potential regional transmission. Genomic analysis revealed the widespread dissemination of ARGs via MGEs, particularly rep plasmids and insertion sequence elements, which contributed significantly to genomic diversity and antibiotic resistance.

**Conclusions:**

This pilot study highlights the clonal diversity of *A. baumannii* in Egypt and underscores the critical role of MGEs in the spread of resistance genes. Targeted genomic surveillance and infection control are essential to curb the spread of high-risk resistant *A. baumannii* clones in Egyptian clinical settings.

**Supplementary Information:**

The online version contains supplementary material available at 10.1186/s12879-025-11185-x.

## Introduction

The 2024 World Health Organization (WHO) Bacterial Priority Pathogens List (BPPL) designates *Acinetobacter baumannii* as one of the top three critical pathogens that pose a serious global public health threat [1]. *A. baumannii* belongs to ESKAPE group that reveals multiple drug resistance (MDR), alongside *Enterococcus faecium*,* Staphylococcus aureus*,* Klebsiella pneumoniae*,* Pseudomonas aeruginos*a and *Enterobacter* spp [2]. As an opportunistic pathogen, *A. baumannii* is implicated in a variety of serious infections, including bloodstream infections, endocarditis, hospital and community-acquired pneumonia, particularly affecting immunocompromised patients [3–11]. Its remarkable ability to survive on inanimate surfaces, such as medical equipment, as well as on the skin of patients, facilitates its persistence and transmission within healthcare settings, leading to frequent nosocomial outbreaks [8].

Carbapenem-resistant *A. baumannii* (CRAB) has emerged as a major public health concern due to its ability to develop resistance to carbapenems, which are often considered one of the last resorts for treating MDR infections [12]. The carbapenem resistance mechanisms in *A. baumannii* are multifaceted, with key contributors including *bla*_OXA−51−like_ genes, which confer low-level carbapenemase activity. Additionally, the overproduction of extended-spectrum β-lactamases (ESBLs) and other β-lactamase genes significantly enhances carbapenem resistance [13–17].

Beyond its antimicrobial resistance (AMR), the pathogenicity of *A. baumannii* is driven by a variety of virulence factors that enable it to thrive in hostile environments. Outer membrane porins are crucial for nutrient uptake and play a role in evading host immune response [18]. Capsular polysaccharides (CPS) form a protective layer around the bacterium, aiding in immune evasion and facilitating biofilm formation. The structural diversity in CPS is driven by genetic variations within the outer core locus (OCL) based on the presence of specific genes, particularly the presence of specific genes such *pda1* or *pda2*. Most CPS synthesis genes are located at the K locus (KL), which is organized into regions responsible for CPS export and structural composition [18–20].

Iron acquisition is another critical virulence mechanism that underscores the adaptability of *A. baumannii*. The bacterium utilizes siderophores like acinetobactin, outer membrane vesicles, and heme acquisition systems to sequester iron from the host. This ability is particularly crucial for survival and virulence under the iron-limited conditions typical of the host environment [21, 22]. Additionally, *A. baumannii* employs a variety of secretion systems (T1SS, T2SS, T4SS, T5SS, and T6SS) to transport and release virulence factors, which play a critical role in its pathogenicity and survival by facilitating host cell adhesion, immune evasion, biofilm formation, and the delivery of toxins that disrupt host cell function [23, 24]. These secretion systems contribute to bacterial fitness, AMR and the persistence of *A. baumannii* in healthcare settings, further complicating infection control and treatment strategies [23, 25, 26].

Moreover, this bacterium evolved mechanisms to withstand heavy metal stress, enhancing its ability to persist in diverse environments, including healthcare settings. Heavy metals such as mercury, arsenic, copper and zinc are frequently used as antimicrobial agents in medical devices and disinfectants. However, *A. baumannii* has developed resistance to these metals, enabling bacterial survival even in the presence of such toxic substances [27–29].

Plasmids play a crucial role in the dissemination of AMR genes within *Acinetobacter* species. These plasmids are uniquely adapted to the *Acinetobacter* genus and are not stably maintained in other Gram-negative bacteria. Conversely, plasmids from other Gram-negative bacteria are rarely found in *Acinetobacter* specie*s* [30]. Notably, plasmids carrying genes encoding serine carbapenemases, particularly OXA-type beta-lactamases, represent the most prevalent mechanism driving carbapenem resistance in *A. baumannii* [31].

Given the rising threat of MDR *A. baumannii*, particularly in LMICs like Egypt, there is a critical need for comprehensive genomic studies to better understand the mechanisms of resistance and virulence [32–35]. Therefore, this pilot study aimed to evaluate the AMR patterns of eighteen non-duplicate *A. baumannii* isolates collected from healthcare facilities in Egypt. Whole-genome sequencing (WGS) was employed to determine sequence types (STs) and international clones (ICs) while investigating genetic determinants associated with AMR and virulence factors. The study also highlighted the diversity of genetic elements and explored chromosomal mutations contributing to AMR and the overall pathogenicity of *A. baumannii* isolates. The insights gained from this study underscore the urgent need for enhanced surveillance, targeted infection control measures, and the development of novel therapeutic strategies to mitigate the spread of multidrug-resistant *A. baumannii* in healthcare settings.

## Methods

### Bacterial isolation, genomic DNA extraction and molecular identification

A total of 18 non-duplicate *A. baumannii* isolates were collected from a clinical microbiology laboratory serving multiple hospitals in Alexandria, Egypt, between August 2020 and April 2021. To maintain diversity and prevent the selection of related isolates, we employed a random sampling approach that considered the month of isolation and source. *A. baumannii* were isolated from different sources, including bronchoalveolar lavage fluid (*n* = 6), wound (*n* = 4), blood (*n* = 3), and sputum (*n* = 2). Additionally, single isolates were recovered from urine, abdominal drain aspirate, and the tip of an abdominal pigtail catheter. As the isolates were collected prior to the study period, ethical approval and patient consent were not required. *A. baumannii* strains were cultured on Brain Heart Infusion Agar (Oxoid, UK), and stock cultures were kept in Brain Heart Infusion Broth with 20% (vol/vol) glycerol at − 80 °C.

Bacterial genomic DNA was extracted using the QIAamp DNA Mini Kit (Qiagen, UK), following the manufacturer’s instructions, as previously described [36]. DNA quality and concentration were assessed using a Nanodrop spectrophotometer (Micro-volume UV-Vis Spectrophotometer FC2100, China). The extracted DNA samples were stored at -20 °C until further use.

A multiplex polymerase chain reaction (PCR) was performed to detect the presence of *bla*_OXA−51_, *bla*_OXA−23_, *bla*_OXA−24_ and *bla*_OXA−58_ genes, with *bla*_OXA−51_ serving as the primary target for the identification of *A. baumannii* [37]. The 25 µl PCR reaction mixture consisted of 12.5 µl PCR Master Mix, 1 µl forward primer, 1 µl reverse primer, 1 µl DNA extract, and 9.5 µl nuclease-free water. PCR conditions were set as follows: initial denaturation at 94 °C for 5 min, followed by 30 cycles of denaturation 94 °C for 25 s, annealing at 52 °C for 40 s, extension at 72 °C for 50 s, and a final extension at 72 °C for 6 min. *A. baumannii* ATCC19606 was used as a positive control for *bla*_OXA−51_.

### Antimicrobial susceptibility testing

Antimicrobial susceptibility testing was performed using the VITEK2 AST-N222 card (bioMérieux; Marcy l’ Etoile, France), following the manufacturer’s instructions. The antimicrobial panel included fourteen antibiotics: ticarcillin, ticarcillin-clavulanic acid, piperacillin, piperacillin-tazobactam, ceftazidime, cefepime, gentamicin, tobramycin, ciprofloxacin, aztreonam, imipenem, meropenem, minocycline, and colistin.

Interpretation of the results was performed according to the Clinical and Laboratory Standards Institute (CLSI, 2024) guidelines [38]. AMR profiles were classified into three categories: MDR: bacteria exhibiting resistance to at least one drug in three different antimicrobial classes; XDR: bacteria showing resistance to most antimicrobials, except for one or two classes; or pan-drug resistant (PDR): bacteria demonstrating resistance to all available antimicrobial agents [39].

### Whole genome sequencing, library preparation, assembly and annotation

The quality of the extracted genomic DNA was further evaluated using a Qubit 3.0 fluorometer (Thermo Fisher Scientific, Waltham, MA, USA). Library preparation was performed using the NEXTflex^®^ Rapid XP DNA-Seq library Preparation kit (Bioo Scientific, Texas, USA). The libraries were sequenced using NextSeq 500/550 kit v2.5 (300 cycles) paired-end kit (Illumina Inc., San Diego, CA, USA). Quality control and adapter trimming of the raw sequencing reads were performed using fastp [40]. De novo genome assembly was performed using Unicycler v0.4 [41], and the quality of draft genome assemblies was assessed using QUAST [42]. Species confirmation was using SpeciesFinder v2.0 (https://cge.food.dtu.dk/services/SpeciesFinder/) [43]. Genomes completeness and contamination were assessed using CheckM with threshold of 90% and 5%, respectively (https://usegalaxy.eu/). Genome annotation was performed using Prokka [44]. Roary software was used to calculate the number of core genes, shell genes, and cloud genes in the isolates pangenome [45]. All sequencing data were deposited under BioProject *PRJNA906141* [46].

### MLST, capsule typing, and mobile genetic element and plasmid analysis

Multilocus sequence typing (MLST) was performed using the *A. baumannii* MLST database on PubMLST. Sequence types were assigned based on both Oxford (*gltA*, *gyrB*, *gdhB*, *recA*, *cpn60*, *gpi*, and *rpoD* genes) and Pasteur (*cpn60*, *fusA*, *gltA*, *pyrG*, *recA*, *rplB*, and *rpoB* genes) schemes available on *pubmlst.org* [47, 48]. Novel allelic profiles following the Oxford scheme were uploaded to PubMLST and assigned new ST^OXF^. The classification of *A. baumannii* global Clones (GCs) or International Clones (ICs) 1, 2, or 3) was performed as previously described [49–52]. Capsular polysaccharide (CPS) typing was performed using Kaptive v2.0.4 [53]. Genetic elements were identified using https://cge.food.dtu.dk/services/MobileElementFinder/.

Col-type plasmids were identified using the MobileElementFinder tool, while replicon clusters (rep-clusters) were detected using MOB-suite v.3.1.0 https://github.com/phac-nml/mob-suite and the SoluGenomics platform https://platform.solugenomics.com/ [54].

### Resistome and virulome characterization

Genome resistome and virulome profiling were conducted using ABRicate https://github.com/tseemann/abricate with underlying databases, including ResFinder Comprehensive Antibiotic Resistance Database (CARD) and Virulence Finder database (VFDB). Redundant genes in the AMR databases were filtered out to ensure accuracy. Resistance genes were categorized by antibiotic class, while virulence genes were grouped based on their associated virulence factors and phenotypes. Additionally, ResFinder online tool (http://genepi.food.dtu.dk/resfinder) was used to detect AMR genes found in filtered reads.

Additionally, chromosomal mutations and single nucleotide polymorphisms (SNPs) associated with AMR were identified using the CARD resistance gene identifier (RGI) tool (https://card.mcmaster.ca/analyze/rgi).

### Phylogenetic tree construction

Core genome multilocus sequence typing (cgMLST) was performed by fast-GeP [55]. Polymorphic core genes (i.e., genes with at least one nucleotide variation across all genomes) were selected and concatenated by the ruby script concat_cgMLST_genes.rb (https://github.com/JoseCoboDiaz/concat_cgMLST_genes). The concatenated gene-by-gene fasta file was used for alignment and phylogenetic tree build using MAFFT version 7 [56] with default parameters. Additionally, Minimum spanning tree (MST) was generated using Ridom SeqSphere + version 9.0.10.

Phylogenetic tree construction was performed using the Neighbor-Joining method with the Jukes-Cantor substitution model and 1000 bootstrap resampling for the construction of the phylogenetic tree. The tree was visualized using iTOL https://itol.embl.de/ [57].

## Results

### General characterization of the isolates

All 18 isolates were confirmed as *A. baumannii* by PCR targeting the intrinsic *bla*_OXA−51_ gene. The genome size of the isolates ranged between 3.7 Mbp and 4.2 Mbp, with an average GC content of 39%. The number of contigs varied among the isolates, with an average of 157 contigs per genome. The average N50 and L50 values were 92,603 bp and 20, respectively. Pangenome analysis of the 18 isolates identified 2007 core genes, 2764 shell genes, and 238 cloud genes, reflecting the genomic diversity among the isolates.

### Phenotypic detection of antimicrobial resistance and carbapenemase production

Using the VITEK2 compact system, all isolates exhibited resistance to most antibiotic classes, including beta-lactams. However, imipenem resistance was detected in only 88.9% (16/18) of the isolates. Resistance to fluoroquinolones was observed in all isolates. Among aminoglycosides, 14 isolates were resistant to gentamicin, while 15 were resistant to tobramycin. Notably, only four isolates exhibited resistance to tetracyclines. Importantly, all isolates were susceptible to polymyxins, with colistin demonstrating 100% efficacy (Table S1). Based on their AMR profiles, 72.2% of *A. baumannii* isolates were classified as XDR, while 26.78% were classified as MDR.

### Clonal distribution and genetic diversity of *A. baumannii* isolates

Based on the MLST analysis using the Pasteur scheme, 15 of the 18 isolates were assigned to 8 distinct STs. The most frequently detected sequence type was ST570^PAS^, accounting for 28% (5/18) of the isolates. ST85^PAS^ and ST2^PAS^ were also identified in 17% (3/18) and 11% (2/18), respectively. The remaining (ST158^PAS^, ST15^PAS^, ST164^PAS^, ST600^PAS^ and ST613^PAS^) were each represented by a single isolate. The three remaining isolates were not assigned to any ST due to missing alleles required to complete the profile (Table S2).

In contrast, MLST analysis using the Oxford scheme identified 11 major STs. ST1701^OXF^ and ST1580^OXF^ were the most prevalent, each detected in three isolates, followed by ST684^OXF^ and ST499^OXF^ were found in two isolates each. Interestingly, three isolates were recently assigned novel STs, including ST3309^OXF^ (*ID18585*) and ST3321^OXF^ (*ID18610* and *ID18611*). Additionally, ST2026, ST1816, ST1418, ST1158 and ST236 were each identified in a single isolate.

Among the identified ICs, IC2 was the most common, observed in 44% (8/18) of the isolates, predominantly linked to ST570^PAS^ and ST2^PAS^. IC9 was present in 17% (3/18) of the isolates, whereas IC5 was detected in 11.1% (2/18). IC4 and IC8 were each found in a single isolate (5.5%). The most prevalent outer core lipooligosaccharide (OCL) type was OCL1, found in 12 isolates (66.7%), followed by OCL5 (22.2%;4/18), while OCL2 and OCL7 were each detected in one isolate (5.5%). Group A comprised OCL1 and OCL2, whereas Group B included OCL5 and OCL7. Regarding capsular (KL) locus typing, KL23 was the most frequently identified, present in 4 isolates (22.2%), followed by KL234 and KL9, each detected in were each found in three isolates (16.67%). KL152 and KL200 were found in 2 isolates each (11.11%), while KL40, KL6, KL4, and KL47 were observed in one isolate each (5.5%) (Table S2).

### Distribution of antimicrobial resistance genes in *A. baumannii*

**Fig. 1:**
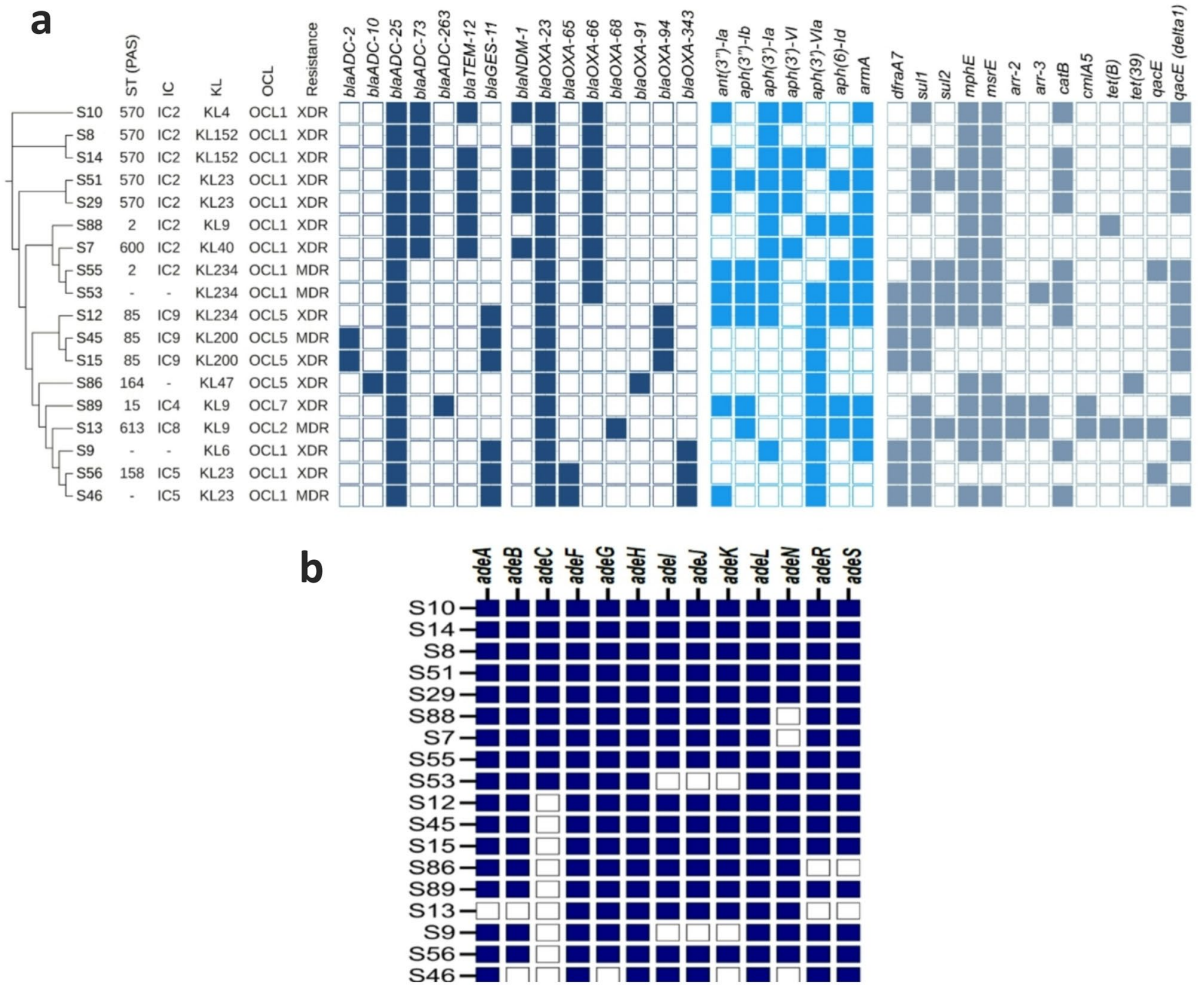
**(a)** cgMLST phylogeny of 18 *A. baumannii* isolates, displaying Pasteur (PAS) multilocus sequence types (MLST), international clones (IC), capsule genes, outer core locus type (OCL), phenotypic resistance patterns, and AMR genes. Colored squares indicate gene presence, while colorless squares indicate absence. **(b)** Prevalence of efflux pump genes among 18 *A. baumannii* isolates. Colored squares indicate the presence of genes, while white squares indicate their absence was performed

In this study, various resistance genes were identified among the isolates (Fig. 1a, Table S2). The prevalence of aminoglycoside resistance genes was as follows: *aac(6’)-Ib* (5/18, 27.8%), *aac(6’)-Ib3* (6/18, 33.3%), *aadA1* (11/18, 61.1%), *ant(3’’)-Ia* (9/18, 50%), *ant(3’’)-IIa* (16/18, 88.9%), *aph(3’)-Ia* (11/18, 61.1%), *aph(3’’)-Ib* (6/18, 33.3%), *aph(3’)-VI* (5/18, 27.8%), *aph(3’)-VIa* (12/18, 66.7%), *aph(6)-Id* (7/18, 38.9%), *armA* (12/18, 66.7%) and *arr-3* (3/18, 16.7%), *arr-2* (2/18, 11.1%).

The *Acinetobacter*-derived AmpC cephalosporinase (ADC) gene variants were identified with the following prevalence: *bla*_ADC-25_ (18/18, 100%), *bla*_ADC-73_ (7/18, 38.9%), *bla*_ADC-30_ (2/18, 11.1%), *bla*_ADC-2_ (2/18, 11.1%), *bla*_ADC-10_, and *bla*_ADC-263_ (1/18, 5.56%). These genes play a significant role in conferring resistance to cephalosporins in *Acinetobacter* species.

Additional beta-lactamase genes in the isolates were as follows: *bla*_CTX−M−14_ was detected in 5.56% (1/18) of the samples, while *bla*_GES−11_ was found in 33.3% (6/18). The carbapenemase gene *bla*_NDM−1_ was identified in 27.8% (5/18) of the isolates. Other *bla*_OXA_ variants, including *bla*_OXA−51_, *bla*_OXA−68_, *bla*_OXA−48_ and *bla*_OXA−91_ were each detected in one out of 18 isolates (5.5%). *bla*_OXA−66_ was found in 9 isolates (50%), while *bla*_OXA−343_ and *bla*_OXA−94_ were each detected in 3 isolates (16.7%). Notably, *bla*_OXA−23_ was the most prevalent variant, present in all isolates (100%).

Additionally, *bla*_PER−7_ was found in 22.2% (4/18), and *bla*_TEM−12_ was observed in 33.3% (6/18) of the samples. The genes *catB8* and *cmlA5*, which provide resistance to chloramphenicol, were detected in 50% (9/18) and 11.1% (2/18) of the isolates, respectively. The gene *dfrA7*, which confers resistance to trimethoprim, was present in 38.9% (7/18) of the samples. Additionally, the genes *mph(E)* and *msr(E)*, involved in resistance to macrolides, were each found in 83.3% (15/18) of the isolates. The genes *tet(39)* and *tet(B)*, which confer resistance to tetracyclines, were detected in 11.1% (2/18) of the isolates. Furthermore, the disinfectant resistance genes *qacE delta 1* and *qacE* were detected in 66.7% (12/18) and 16.7% (3/18) of the isolates, respectively. The sulfonamide resistance genes *sul1* and *sul2* were found in 77.8% (14/18) and 27.8% (5/18) of the samples, respectively. In addition, genes associated with heavy metal resistance were identified: for arsenic, *arsA* was present in 11.1% (2/18), *arsB* in 16.7% (3/18), and *arsC* in 16.7% (3/18) of the isolates. For mercury, *merA*,* merR*, and *merT* were each detected in 5.5% (1/18), while *merD* was found in 11.1% (2/18) of the isolates (Table S2).

Interestingly, multiple families of efflux pumps were detected, including resistance-nodulation-cell division (RND), small multidrug resistance (SMR), multidrug and toxic compound extrusion (MATE), and major facilitator superfamily (MFS). These efflux systems confer resistance against nearly all classes of disinfectants and antimicrobials, including carbapenems. Efflux pump genes were detected in almost all isolates (Fig. 1b, Table S6), with the RND family being the most prevalent. Notably, *adeF*, *adeH* and *adeL* were present in all the isolates. Additionally, all isolates harbored *abeM* and *abeS*, belonging to the MATE and SMR families, respectively.

### Diversity of beta-lactamase genes among international clones

A strong association was observed between detected antimicrobial resistance genes (ARGs) and IC. Specifically, the *bla*_OXA−66_ gene was exclusively detected in IC2, while *bla*_OXA−65_ was associated with IC5. Among IC2 isolates, *bla*_ADC−73_ was prevalent (87.5%), except in ST33021^OXF^/ ST2^PAS^, whereas *bla*_ADC−263_ was restricted to IC4. Additionally, *bla*_OXA−68_ was confined to IC8, and *bla*_OXA−94_ was exclusively found with IC9, demonstrating the distinct distribution of β-lactamase genes among different clonal lineages (Fig. 1a, Table S2).

### Association of plasmids and mobile genetic elements (MGEs) with ARGs

**Fig. 2:**
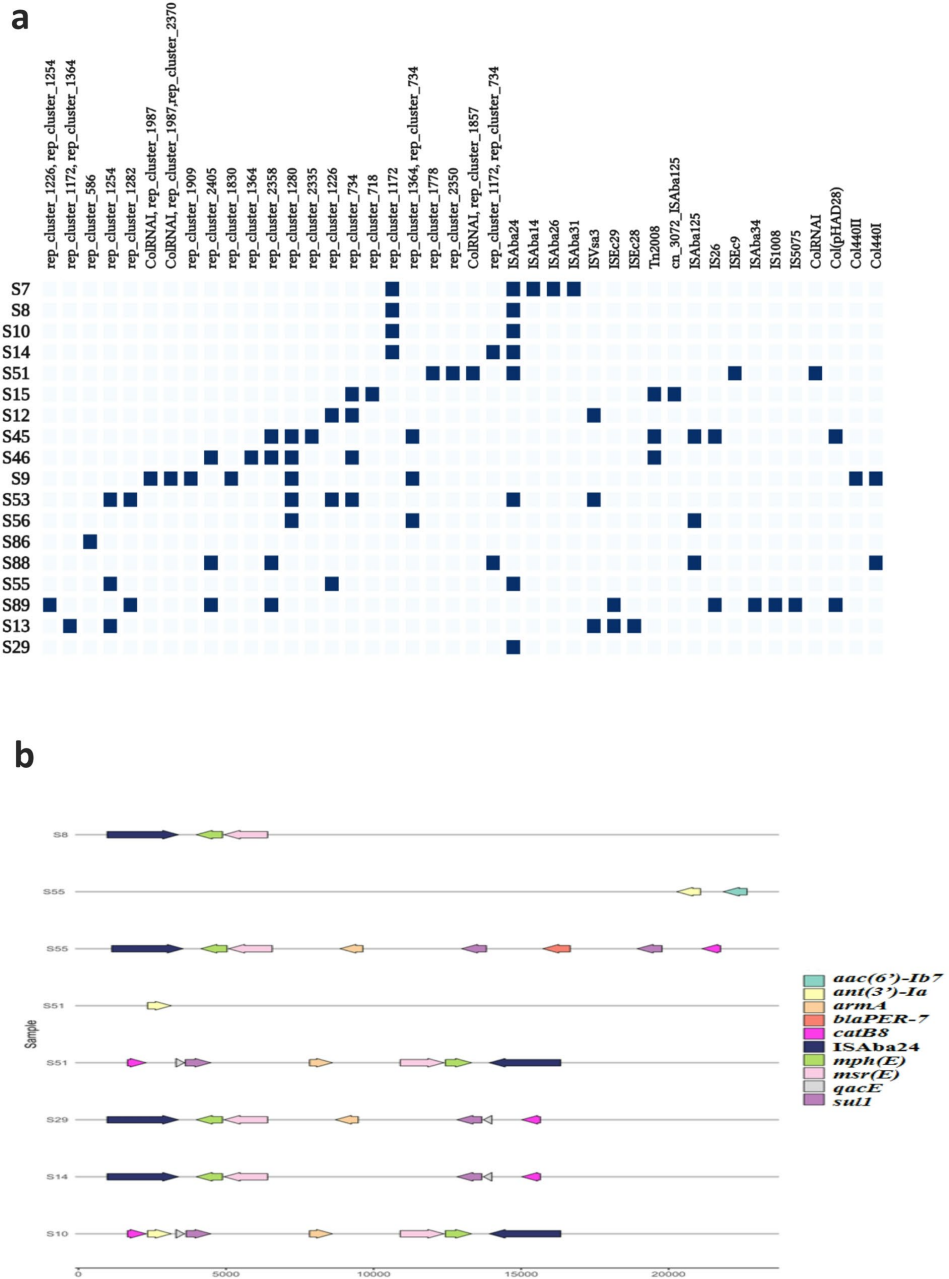
**(a)** Distribution of genetic elements among isolates, including insertion sequences, rep clusters- and Col plasmids. Navy color indicates the presence of these elements, while baby blue represents their absence. **(b)** Flanking AMR genes around ISAba24

In silico analysis revealed a diverse repertoire of insertion sequences and plasmid replicons, including Col and rep types, harboring ARGs in *A. baumannii* isolates. ColRNAI and Col440II were detected in one isolate, whereas Col440I and Col(pHAD28) were identified in two isolates (Fig. 2a, Table S5). Additionally, rep types exhibited an average size of 33276.9 bp and an average GC content of 40.29%. A total of 33 rep-type plasmids were classified into mobilizable (40%), conjugative (35%), and non-mobilizable (25%) categories. The most frequently observed clusters included rep_cluster_1172 and rep_cluster_734, which were associated with multiple mobility types.

A wide array of identified AMR genes was linked to specific rep types. For instance, *bla*_*OXA−23*_ was consistently associated with rep_cluster_1172, either alone or in combination with rep_cluster_734. Several resistance genes, including *aac(6’)-Ib*,* aadA1*,* aph(3’)-Ia*,* armA*,* catB8*,* mph(E)*,* msr(E)*,* qacE delta1* and *sul1*, were found without any associated rep type. However, genes such as *bla*_*GES−11*_, *bla*_*OXA−23*_ and *dfrA7* were associated with rep_cluster_734 or combinations such as rep_cluster_1364/rep_cluster_734. Similarly, specific resistance patterns were observed, such as *bla*_*PER−7*_, *msr(E)*,* mph(E)*, and *armA* were linked to rep_cluster_1254, while other combinations included rep_cluster_1226, rep_cluster_1280, and rep_cluster_586. (Fig. 2a, Table S5)

Beyond plasmid replicons, various insertion sequences (ISs) and transposons were identified in the isolates, contributing to the dissemination of antimicrobial resistance determinants. The most frequently detected IS elements were: ISAba24 (8/18), ISAba125 (3/18), ISVsa3 (3/18), IS26 (2/18), ISEc29 (2/18), ISAba14 (1/18), ISAba26 (1/18), ISEc9 (1/18), IS5075 (1/18), IS1008 (1/18), ISAba34 (1/18), ISEc28 (1/18), and ISAba31 (1/18). Notably, all IC2 carried ISAba24, except for one isolate (Fig. 2a, Table S3).

Additionally, several transposons associated with resistance determinants were detected, including cn_3072_ISAba125 and Tn2008. *bla*_OXA−23_ was found within Tn2008 in three isolates. Interestingly, the novel ST3321^OXF^, found in two isolates, carried ISAba24 and carried specific ARGs such as *msrE*, *mphE* and *armA*, further emphasizing the role of MGEs in the horizontal transfer of resistance genes (Fig. 2b).

### Chromosomal mutations

All the isolates exhibited 100% resistance to fluoroquinolones due to antibiotic target alteration. Mutations in the quinolone resistance-determining regions (QRDRs) of the *gyrA* and *parC* genes were identified, including SNP S81L in *gyrA*, and mutations at S84L, V104I and D105E in *parC.* Furthermore, a mutation in the bacterial elongation factor Tu (*EF-Tu)* gene (R234F) is associated with elfamycin resistance. This mutation was specifically observed in isolate S89 (ST236^OXF^/ST15^PAS^).

### Virulome analysis of *A. baumannii*

Analysis of all *A. baumannii* isolates using the Virulence Factor Database (VFDB) identified a diverse set of genes associated with pathogenicity and virulence. Iron acquisition genes, including *entE*,* barA*,* barB*,* basA-J*,* bauA-F* and *hemO* cluster genes, were present across the isolates. CPS biosynthesis genes, such as *galE*,* galU*,* pgi* and *tviB*, were detected in most isolates, with some variation in the presence of *pse* genes. Lipopolysaccharide (LPS) biosynthesis genes, including *lpsB*,* lpxA-D*,* lpxL*, and *lpxM*, were consistently identified in all isolates. The outer membrane protein *ompA* was present in all isolates. Additionally, secretion system genes were widely detected, with Type II Secretion System (T2SS) genes (*gspC-M*) and Type VI Secretion System (T6SS) genes (*tssA-M*,* hcp/tssD*,* clpV/tssH* and *vgrG/tssI*) found in most isolates. These findings suggest a conserved virulence gene profile, supporting the pathogenic potential and adaptability of the isolates (Table S4).

### Core genome MLST characterization

The cgMLST scheme was used to investigate the relatedness of *A. baumanii* isolates assigned to ST570^PAS^ and ST85^PAS^ with globally reported isolates available on Pathogen watch https://pathogen.watch/.

**Fig. 3:**
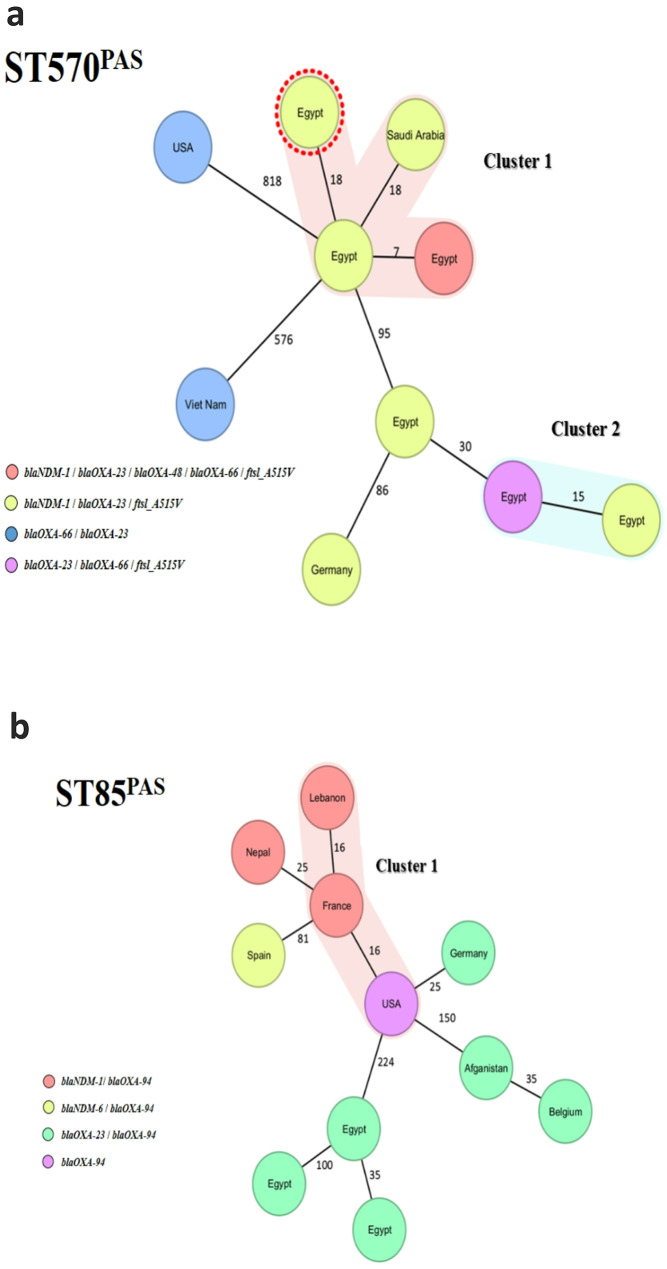
**(a)** Minimum spanning tree (MST) generated using Ridom SeqSphere + to illustrate 10 ST570 *A. baumannii* isolates, which include 6 Egyptian and 4 worldwide isolates. The cluster distance threshold is set at 20. Each node is labeled with the country of isolation and color-coded according to the presence of carbapenem resistance genes. The ST570 isolate from outside Egypt is marked with a red circle. **(b)** Minimum spanning tree (MST) generated using Ridom SeqSphere + illustrating 11 ST85 *A. baumannii* isolates, which include 3 Egyptian and eight worldwide isolates. The cluster distance threshold is set at 20. Each node is labeled with the country of isolation and color-coded according to the presence of carbapenem resistance genes

For ST570^PAS^, five publicly available carbapenem-resistant *A. baumannii* genomes of clinical origin were selected, with only one isolate per country included (Table S7). cgMLST and phylogenetic reconstruction analysis revealed two distinct clusters, including our Egyptian isolates (Fig. 3a). The larger Cluster 1 compressed three Egyptian isolates closely clustering with a clinical isolate from Saudi Arabia, likely due to geographical proximity. Nevertheless, the Egyptian *A. baumannii* isolates showed no genetic relatedness to other ST570^PAS^ isolates from the USA, Vietnam, or Germany (Fig. 3a). For ST85^PAS^, eight publicly available genomes of carbapenem-resistant *A. baumannii* isolates of clinical origin were selected, again with only one isolate per country included (Table S8). cgMLST analysis identified 1 clonal cluster containing isolates from Lebanon, France and the USA. However, the Egyptian isolates revealed no clustering together or with any other isolates from different countries (Fig. 3b).

## Discussion

The increasing prevalence of CRAB infections poses a serious public health concern, as the bacterial species exhibits MDR and a remarkable ability to acquire additional resistance genes and chromosomal mutations [58–60]. The Middle East, particularly Egypt, reports the highest reported prevalence of CRAB, with immunocompromised patients and those in intensive care units (ICUs) being the most at risk [35, 61–64]. Therefore, this pilot study investigated the AMR profiles of eighteen *A. baumannii* isolates from Egyptian healthcare facilities and assessed clonal relationships using cgMLST and MLST-based typing (Pasteur and Oxford Schemes), along with virulence factors, resistance determinants, and mobile genetic elements involved in resistance dissemination. Phenotypic screening revealed that the majority of the isolates were resistant to carbapenem, fluoroquinolones and aminoglycosides, classifying 72.2% as XDR and 26.78% as MDR. These findings align with previous reports highlighting the high prevalence of XDR *A. baumannii* worldwide and in Egypt [35, 64–66]. Notably, colistin remains the last-resort treatment against CRAB infections [67, 68]. Given that colistin is one of the few remaining effective options, the increasing reliance on it raises serious concerns regarding the potential emergence of colistin resistance, which could further restrict therapeutic choices and complicate infection management in critical care settings. In this study, all *A. baumannii* isolates were susceptible to colistin. However, a recent study [69] reported that 12.9% of *A. baumannii* isolates were phenotypically resistant to colistin, and harbored novel mutations in several outer membrane proteins, in addition to previously described mutations in *pmrB*, underscoring the evolving genetic mechanisms underlying colistin resistance [70].

This study reinforces the critical role of OXA-type carbapenemases, particularly *bla*_OXA−51_ and *bla*_OXA−23_, in driving β-lactam resistance in *A. baumannii*, and highlights the urgent need for continuous molecular monitoring and the exploration of alternative therapeutic options, consistent with previous reports identifying these enzymes as the most prevalent carbapenemases in *A. baumannii* [62, 71–73]. Other detected β-lactamase genes included *bla*_OXA−58_ and *bla*_OXA−24/40,_ all contributing to resistance against β-lactam antibiotics [74, 75].

The clonal dominance of high-risk clones observed in this study, particularly the identification of ST570^PAS^/ ST1701^OXF^ belonging to IC2, a well-documented MDR lineage with global dissemination potential [65, 76] that has been reported to be associated with hospital-acquired infections worldwide [77–79], including Egypt [35, 80–82], This was followed by the detection of IC5 and IC9, which have also been reported in Northern Africa, particularly Egypt [32]. The widespread presence of these international clones among the screened isolates reflects the progressive expansion and establishment of high-risk lineages in the local clinical settings. The study further revealed correlations between β- lactamase genes and international clones, such as *bla*_OXA−66_ in IC2, *bla*_OXA−343_ in IC5, *bla*_ADC−6_ in IC4, *bla*_OXA−68_ in IC8, and *bla*_OXA−94_ in IC9, reinforcing previous reports on the distribution of these resistance genes [32, 83, 84]. The cgMLST-based clustering patterns of ST570^PAS^ isolates underscore the localized evolution and potential regional transmission dynamics of *A. baumannii* in Egypt and neighboring Countries. The clear genetic separation between Egyptian isolates and those from more distant regions highlights the limited global dissemination of these strains and suggests that local clonal expansion, rather than widespread international spread, is currently driving their circulation. These findings highlight the need for enhanced regional surveillance and infection control to control the spread of CRAB.

In addition to AMR, the screened isolates also exhibited the presence of heavy metal resistance genes, which may contribute to their persistence in hospital environments and other contaminated settings. The co-selection of antimicrobial and heavy metal resistance is a growing concern, as exposure to heavy metals in clinical and environmental settings may promote the maintenance and spread of MDR strains [85]. Consistent with previous studies [86–88], heavy metal resistance plays a crucial role in enhancing bacterial survival under harsh conditions. This also highlights the significance of MGEs in the dissemination of heavy metal resistance, facilitating its spread within bacterial populations.

The presence of efflux pump genes was detected in all isolates, highlighting their critical role in AMR in *A. baumannii*, and reinforces the need to explore efflux pump inhibitors as potential complementary therapies to restore antimicrobial efficacy against resistant strains. Notably, *abeM* (MATE family), *abeS* (SMR family), *amvA* (MFS family), and *adeA*, *adeC*, and *adeG* (all belonging to the RND family) were widely distributed among the isolates, supporting previous findings that emphasize the widespread presence and functional diversity of these efflux systems in *A. baumannii* [89]. By actively extruding a broad range of antibiotics, these pumps lower intracellular drug concentrations, enhance bacterial survival during treatment, and contribute significantly to the development of multidrug resistance [90–92].

Our study also uncovered key chromosomal mutations associated with AMR. Specifically, mutations in DNA gyrase (*gyrA*-S81L) and topoisomerase IV (*parC*-S84L, V104I and D105E) were linked to fluoroquinolone resistance, consistent with previous report [93]. Additionally, a mutation in elongation factor Tu (*EF-Tu*- R234F) was identified, which has been associated with resistance to elfamycin and impaired bacterial translation. A similar mutation has been reported in *E. coli*, suggesting a conserved mechanism of resistance across different bacterial species [94]. The recurrence of analogous mutations across bacterial species suggests a conserved evolutionary pathway for resistance development, underscoring the critical need for high-resolution genomic surveillance and functional validation studies to anticipate the emergence of resistance and optimize therapeutic strategies.

Although short-read sequencing is known to have limitations in assembling transposons, a diverse array of insertion sequences and transposons was identified among the screened isolates, including ISAba24, ISAba125, ISAba14, ISAba26, IS26, ISEc9, IS5075, IS1008, ISAba34, ISVsa3, ISEc29, ISEc28, ISAba31, cn_3072_ISAba125, and Tn2008. These transposon-dependent gene transfers were observed across different *A. baumannii* clones, highlighting their widespread presence and suggesting a major role in the dissemination of carbapenem resistance genes. The presence of ISAba24 in two isolates, co-localized with *msrE*, *mphE* and *armA*, highlights its potential role in facilitating the spread of macrolide and aminoglycoside resistance genes and underscores the significant role of this element in mobilizing macrolide and aminoglycoside resistance genes in *A. baumannii*.

Additionally, the detection of Col plasmids from diverse subtypes, including Col (pHAD28), Col440I, Col440II and ColRNAI, suggests a role in the dissemination of resistance genes. Consistent with previous reports [95–98], Col plasmids such as Col (pHAD28), ColRNAI, Col440II, and Col440I were identified in *Acinetobacter baumannii*, *Klebsiella pneumoniae*, *Escherichia coli*, and *Salmonella enterica*, highlighting their role in facilitating horizontal gene transfer (HGT) across diverse bacterial species.

Virulome analysis revealed that all isolates carried genes involved in bacterial adhesion, biofilm formation, iron acquisition, and toxin production. The predominance of capsular polysaccharide (CPS) genes, particularly OCL1, was associated with IC2, aligning with previous findings [83, 99]. The presence of iron acquisition genes, such as *barB*,* basA-J*,* bauA-F* and the *hemO* cluster, across all isolates, highlights their crucial role in iron uptake and virulence. These genes likely help the isolates overcome iron limitation, thereby enhancing their adaptability and pathogenicity. Similar findings have been reported in previous studies, where these genes were associated with improved survival in iron-restricted conditions [21, 100, 101]. The widespread distribution of virulence-associated loci across genetically diverse CRAB isolates underscores their enhanced fitness and pathogenic potential, particularly in the nosocomial niche, where selective pressures such as antibiotic exposure and host immune compromise facilitate the persistence, transmission, and outbreak potential of these highly adapted strains. This convergence of resistance and virulence factors not only complicates treatment options but also increases the risk of prolonged hospital outbreaks, severe infections, and higher morbidity and mortality rates [102, 103].

The presence of CPS biosynthesis genes, including *galE*,* galU*,* pgi* and *tviB*, in most screened isolates suggests their potential role in capsule formation, which contributes to immune evasion and bacterial persistence. However, the variability in *pse* gene presence may indicate genetic diversity in capsular composition among the isolates. Additionally, the consistent detection of LPS biosynthesis genes, such as *lpsB*,* lpxA-D*,* lpxL*, and *lpxM*, highlights the essential role of LPS in outer membrane integrity and pathogenicity. These findings are consistent with previous studies linking CPS and LPS biosynthesis genes to enhanced bacterial survival and virulence within host environments [104,105,106,107].

The presence of the outer membrane protein *ompA* in all isolates highlights its conserved role in membrane integrity, adhesion, and immune evasion. Additionally, the widespread detection of secretion system genes suggests their significance in host interactions and environmental adaptation. That aligns with previous studies that have associated these genes with bacterial virulence and competitive advantages in diverse environments [18, 24, 107–109]. In particular, T2SS genes (*gspC-M*) and T6SS genes (*tssA-M*,* hcp/tssD*,* clpV/tssH*, and *vgrG/tssI*) were identified in most isolates, indicating their potential involvement in protein secretion, virulence, and interbacterial competition. These findings are consistent with previous studies linking T2SS and T6SS to enhanced bacterial survival and pathogenicity [23, 110].

## Conclusion

This pilot study offers preliminary insights into the clonal distribution and potential transmission dynamics of *Acinetobacter baumannii* in Egyptian healthcare settings. The data suggest that horizontal gene transfer and mobile genetic elements (MGEs) may play important roles in shaping the resistance landscape of circulating strains. These mechanisms could contribute to the bacterium’s capacity to adapt to antibiotic pressure, posing ongoing challenges for infection control and treatment. While the findings are limited by the sample size, geographic coverage, and scope of the study, they point to the need for broader genomic surveillance, strengthened antimicrobial stewardship, and exploration of alternative therapeutic strategies to address the rising threat of antimicrobial resistance.

## Electronic supplementary material

Below is the link to the electronic supplementary material.

Supplementary Material 1

## Data Availability

Raw reads of the samples analyzed in this study were deposited in Sequence Read Archive and BioProject NCBI database under the accession number PRJNA906141. BioSample accessions and SRA accessions are available in Additional File 1 Supplementary Table S7.

## Abbreviations

AMR: Antimicrobial resistance
ARG: Antimicrobial resistance gene
CARD: Comprehensive Antibiotic Resistance Database
cgMLST: Core genome multilocus sequence typing
CPS: Capsular polysaccharides
CRAB: Carbapenem-resistant Acinetobacter baumannii
ESBL: Extended-spectrum β-lactamase
GC: Global clone
IC: International clone
IS: Insertion sequence
MDR: Multiple drug-resistant
MGE: Mobile genetic element
MLST: Multilocus sequence typing
OCL: Outer core lipooligosaccharide
PCR: Polymerase chain reaction
PDR: Pan drug-resistant
ST: Sequence type
VFDB: Virulence Finder database
WGS: Whole-genome sequencing
XDR: Extensively drug-resistant
